# Oligodendrocyte Development and Regenerative Therapeutics in Multiple Sclerosis

**DOI:** 10.3390/life11040327

**Published:** 2021-04-09

**Authors:** Nadjet Gacem, Brahim Nait-Oumesmar

**Affiliations:** Institut du Cerveau—Paris Brain Institute—ICM, Inserm, CNRS, AP-HP, Hôpital de la Pitié Salpêtrière, Sorbonne Université, 75013 Paris, France

**Keywords:** oligodendrocytes, development, myelination, multiple sclerosis, remyelination

## Abstract

Myelination by oligodendrocytes (OLs) is an important biological process essential for central nervous system (CNS) development and functions. Oligodendroglial lineage cells undergo several morphological and molecular changes at different stages of their lineage progression into myelinating OLs. The transition steps of the oligodendrocyte progenitor cells (OPCs) to myelinating oligodendrocytes are defined by a specific pattern of regulated gene expression, which is under the control of coordinated signaling pathways. Any abnormal development, loss or failure of oligodendrocytes to myelinate axons can lead to several neurodegenerative diseases like multiple sclerosis (MS). MS is characterized by inflammation and demyelination, and current treatments target only the immune component of the disease, but have little impact on remyelination. Recently, several pharmacological compounds enhancing remyelination have been identified and some of them are in clinical trials. Here, we will review the current knowledge on oligodendrocyte differentiation, myelination and remyelination. We will focus on MS as a pathological condition, the most common chronic inflammatory demyelinating disease of the CNS in young adults.

## 1. Introduction

Oligodendrocytes (OLs) are the myelinating cells in the central nervous system (CNS) [1]. They are generated after an orchestrated process of specification (switch of neural progenitors’ cells from producing neuronal to glial cells), proliferation, migration and differentiation during brain, optic nerve and spinal cord development [2]. The formation of compact myelin provides insulation for axons and allows the saltatory conduction of action potentials, which is necessary to ensure proper neuronal functions. Furthermore, several studies showed that oligodendrocytes provide to neurons essential metabolic and trophic supports [3,4,5,6]. During CNS development, OLs are derived from oligodendrocyte progenitor cells (OPCs). OPCs also persist in the adult CNS, where they represent 5% to 8% of total glial cells and are widely distributed in white matter (WM) and to a less extent in grey matter (GM) [7]. This small fraction of adult OPCs remains in a slowly proliferative quiescent state, contributes to myelin remodeling under physiological conditions and to remyelination following a demyelinating injury. Here, we reviewed current knowledge on OPC specification and differentiation into myelinating OLs in physiological conditions and in multiple sclerosis (MS). We also discussed the relevance of these mechanisms for the development of therapeutic approaches for demyelinating diseases.

## 2. Oligodendrocyte Development

### 2.1. Emergence and Specification of Oligodendrocyte Progenitor Cells (OPCs)

**Figure 1:**
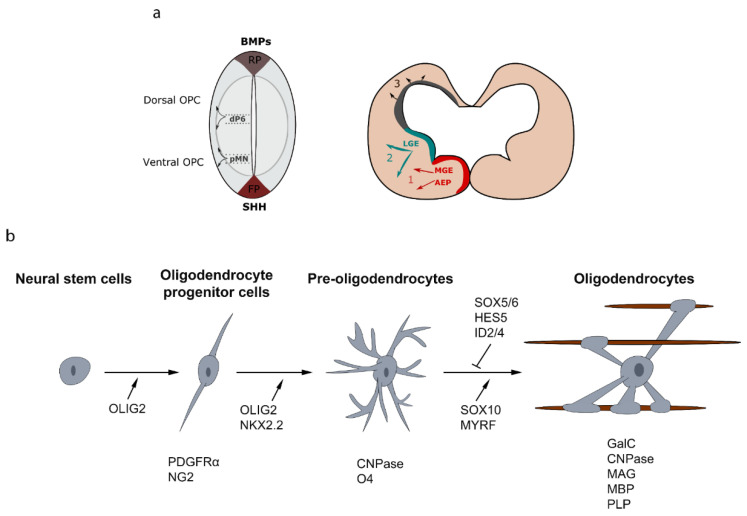
Developmental sources of oligodendrocyte progenitor cells (OPCs) and oligodendroglial cell lineage. (**a**) Sections of spinal cord and telencephalon showing the successive waves of oligodendrogliogenesis at different developmental stages. (**b**) Specification and differentiation of neural stem cells into myelinating oligodendrocytes are characterized by distinct stages, which can be identified by the acquisition and/or the disappearance of several specific markers like platelet-derived growth factor receptor (PDGFR)-α, O4 and proteolipid protein (PLP). The oligodendrocyte lineage progression is under the control of specific transcriptional factors. RP: roof plate, FP: floor plate, AEP: entopeduncular area, MGE: medial ganglionic eminence, LGE: lateral and caudal ganglionic eminences.

Significant progress has been made to discern the signaling pathways implicated in oligodendrocyte specification and differentiation, both in normal and pathological conditions. In the brain, the determination of OPCs from neural progenitors occurs in several waves from multiple germinative ventricular zones of the ventral and dorsal forebrain [8,9]. During embryonic development of the telencephalon, a first wave of OPCs is generated from Nkx2.1+ (NK2 Homeobox 1) precursor cells in the entopeduncular area (AEP) and the medial ganglionic eminence (MGE) at embryonic day 12.5 (E12.5) [10,11]. This first wave of OPCs is followed by a second wave from Gsh2 (GS homeobox 2)-expressing precursors of the lateral and caudal ganglionic eminences (LGE-CGE) at E15.5. Finally, a third wave of OPCs is generated after birth from Emx1+ (Empty spiracles homeobox 1) precursor cells of the subventricular zones (SVZ) lining the lateral ventricles. Strikingly, the earliest OPCs emerging from the MGE-AEP, are completely eliminated, during postnatal development, in most part of the brain and replaced by the two later waves, except in the ventral forebrain and corpus callosum [8,9,12]. Cortical OPCs continue to be generated postnatally from precursor cells located in the SVZ, and migrate radially out of the SVZ to colonize the entire WM and cortex [13,14,15]. In the spinal cord, OPC specification occurs at around E12.5 in the precursor motoneuron (pMN) domain. These ventrally derived OPCs give rise to 85–90% of total populating oligodendrocytes. This first wave of OPCs is followed by a second wave from the dorsal ventricular domain dP6 at E14.5 and contribute to 10–15% of total myelinating oligodendrocytes [16,17,18] (Figure 1a).

In these different regions, the generation of OPCs is under the control of several intrinsic and extrinsic factors. During development, one of the well-established effectors is the morphogen Sonic Hedgehog (Shh), secreted from the notochord and floor plate, which has been largely implicated in oligodendrogliogenesis in ventral ventricular zones. Shh regulates the expression of key transcription factors implicated in OPC specification including Olig2 and Nkx2.2 [19]. Recently, the use of conditional knockout mice showed also that highly regulated Shh signaling from multiple embryonic sources is required for oligodendrocyte generation from dorsal forebrain progenitors in neocortical regions. For their late embryonic transition to oligodendrogenesis, interneurons migrating into the neocortex and the choroid plexus are important sources of Shh ligand [20]. These studies showed also that some population of dorsal oligodendroglial cells are more affected by the loss of Shh signaling than others [21]. In addition to Shh, fibroblast growth factors (FGFs), bone morphogenetic proteins (BMPs) and Wingless-related integration site (WNTs) provide crucial signaling to form a morphogen gradient along the dorso-ventral axis of the CNS [22].

After their specification, OPCs migrate from their birthplaces to reach their final destinations. Interestingly, several studies showed that distinct OPC populations have the capacity to recover the depletion of oligodendrocytes. For instance in the brain, a specific deletion of dorsally derived OPCs induces a transient reduction in oligodendrocyte number, which is recovered by the expansion of ventrally derived OPCs [8,21]. Moreover, after a specific ablation of ventrally-derived OPCs in the spinal cord, dorsally-derived OPCs are able to undergo a rapid amplification, to colonize the ventral spinal cord and to myelinate axons, although with a temporal delay [23]. These data indicate that ventrally- and dorsally-derived OPC populations have the capacity to compensate the loss of each other, suggesting their functional redundancy.

### 2.2. Differentiation of OPCs into Oligodendrocytes

During development, OPCs undergo morphological and signaling changes to differentiate into myelinating oligodendrocytes. Each step is characterized by the acquisition or/and the disappearance of several specific markers. OPCs express platelet-derived growth factor receptor (PDGFR)-α that stimulates their migration and proliferation and inhibits differentiation. Another marker for OPCs is the proteoglycan NG2. During their transition to pre-oligodendrocytes, OPCs change their morphology and acquire a large cell body and more extended branching. At this stage, they express the cell surface markers O4 and 2′,3′-cyclic nucleotide 3′-phosphodiesterase (CNPase) [24,25]. Then, pre-oligodendrocytes give rise to differentiated and mature oligodendrocytes that are characterized respectively by the expression of galactocerebroside (GalC)/O1 and myelin proteins, such as the myelin basic protein (MBP), the myelin associated glycoprotein (MAG) and the transmembrane protein proteolipid protein (PLP). The different stages of the oligodendroglial lineage cells and their main markers are illustrated in Figure 1b.

The development of the oligodendroglial lineage cells into myelinating glia is a complex process regulated by several mechanisms and signaling pathways that control a series of transcription factors. For instance, the basic helix-loop-helix (bHLH) transcription factor Olig2 is required for OPC specification during CNS development. Indeed, Olig2 inactivation lead to a depletion of oligodendrocytes [26,27], while Olig2 overexpression promotes the generation of OPCs [28]. The closely related bHLH transcription factor Olig1 has a minor role in OPC commitment and differentiation. In fact, Olig1 null mice show only a slight oligodendrocyte differentiation delay and has no obvious myelination defects [29,30]. Sox10 (Sry-related HMg-Box gene 10) and Nkx2.2, which are induced by Olig2, act in parallel to control oligodendrocyte differentiation and are essentials for myelin gene expression [31]. Sox 10 is a specific marker of oligodendrocyte lineage in the CNS and is a key regulator of oligodendrocyte differentiation and myelination by regulating several genes implicated directly in this process, including the transcription factor Myrf (myelin regulatory factor) [32,33]. Another HMG-box transcription factor, Sox17, was identified as a positive or negative regulator of oligodendrocyte development, depending on the oligodendroglial cell stage. Sox17 over-expression increase the pool of OPCs during early postnatal development and acts as a negative regulator of OPC differentiation and myelination [34]. While, inactivation of Sox17 reduces the number of Olig2 positive cells and, as a consequence, the number of mature oligodendrocytes [35]. Moreover, Sox5 and Sox6 regulate oligodendrocyte development in the mouse spinal cord. These group D of Sox transcription factors inhibits OPC specification and terminal differentiation [36]. Hes1/5 and Id2/4 were also identified to maintain cells at the OPC stage and inhibits their differentiation [37,38]. Nuclear receptors form another class of transcription factors implicated in oligodendrocyte lineage development and differentiation, and act through dimerization. This family of transcription factors include LXR (liver X receptors), RXR (retinoid X receptors), PPAR (peroxisome proliferator-activated receptors), vitamin D receptors, and THR (thyroid hormone receptors). They are activated through homodimerization or dimerization and can bind to their co-repressors or co-activators. Several studies demonstrated that these nuclear receptors are important for the transcriptional control of myelin gene expression and play key roles in oligodendrocyte differentiation and myelination processes. For example, knocked out mice for LXRs exhibited thinner myelin sheaths and reduced myelin gene expression [39]. The main function of LXRs is to coordinate lipid metabolism, essential for the myelin production by oligodendrocytes. Moreover, deficient mice for RXR-γ showed delayed differentiation into mature oligodendrocytes [40]. It was also reported that the vitamin D receptor forms a heterodimer with RXR and induces OPC differentiation [41].

Axonal signals, among which action potentials, also regulate the lineage progression from OPCs to myelinating oligodendrocytes. Indeed, compelling evidence indicates that oligodendroglia cells are able to sense neuronal activity [42,43] and preferentially myelinate electrically active axons. Action potentials along axons trigger neurotransmitter vesicular release that is a key modulator of the myelination process [44,45,46]. Interestingly, OPCs are also synaptically innervated by glutamatergic and GABAergic neuronal fibers throughout the CNS [47,48]. These synaptic communications between axons and OPCs may constitute a possible mechanism to control oligodendrocyte development in an activity-dependent manner. However, the functional role of these synaptic connections in myelination has not yet been clearly established in vivo.

### 2.3. Regulation of Central Nervous System (CNS) Myelination

The maturation of oligodendrocytes into myelinating cells pass through different morphological changes and is characterized by expression of oligodendrocyte-specific proteins and lipids. When oligodendrocytes start to mature, they produce a multilayered myelin sheath [49]. Myelin contains 70–80% of lipids and around 30% of proteins, among which MBP and PLP are the most abundant. An oligodendrocyte myelinates several internodes located on different nerve fibers in the CNS and that terminate at paranodal loops bordering the nodes of Ranvier [50]. In addition to the main transcription factors listed above, several others have been implicated in the myelination process. These proteins are regulating gene expression and act directly by stimulating several genes controlling myelination, such as Olig2, Sox10, Yy1 (Yin and Yang 1), Nxk2.2 and Myrf, or indirectly by repressing repressors of myelination [51]. The signaling pathways regulating myelination include the phosphoinositide 3-kinase (PI3-kinases)/Akt/ mammalian target of rapamycin (mTOR) and extracellular signal-regulated kinases (ERK)-1/2 pathways, which have a fundamental role in oligodendrocyte development, myelination, and remyelination [52,53,54,55]. The Wnt pathway has been also well characterized and is largely implicated in oligodendrocyte development. Studies suggest that Wnt/β-catenin pathway activation impaired myelination [56].

Axons express also on their surface inhibitors and molecules permissive of myelination. This mechanism controls the need or not of myelination, to ensure correct neuronal functions. Interestingly, most of these molecules are identified as inhibitors of myelination. These inhibitory axonal signals such us Jagged (acting via Notch signaling), PSA-NCAM, and LINGO-1, activate various transcriptional regulators such as Hes5, Sox 5/6, and Id2/4, and therefore prevent OPC differentiation into myelinating oligodendrocytes [57,58]. Other transcription factors, such as sterol regulatory element binding proteins (SREBPs), regulate the expression of genes involved in lipid synthesis, which is crucial for a proper myelination. Indeed, recent studies suggest that a substantial fraction of the lipids incorporated during myelin formation is from astrocytes in the normal brain. Lipid metabolism via SREBPs signaling in astrocyte supply myelin formation in oligodendrocytes through transcellular transport. SREBPs expression and lipid metabolism and storage are controlled by the mTOR pathway, which is also essential for myelination [59,60].

Myelination occurs also in the adult CNS in response to neuronal activity or environmental cues [61,62], and contributes to the slow turnover of the myelin sheaths caused by the loss and dysregulation of OLs occurring during aging [63]. For that, OPCs persist in the adult and tend to homeostatically maintain their cell number [64]. Cell fate mapping [65] and transplantation experiments of adult OPCs [66], as well as RNAseq studies clearly demonstrated that the myelinating capacity of adult OPCs are not similar in all regions [67,68]. For instance, adult OPCs in the CNS white matter have higher proliferation and differentiation rate into myelinating oligodendrocytes than those located in the gray matter, where they remain mainly undifferentiated under physiological conditions.

Spontaneous remyelination of demyelinated axons is an important mechanism that occurs in pathological conditions. The different steps of the remyelination process include OPC activation/proliferation, their migration towards the demyelinated area and finally their differentiation into myelinating oligodendrocytes [69]. These steps are highly regulated by several mechanisms that recapitulate partially those involved during developmental myelination, such as nuclear receptors and their ligands (LXRs α/β, PPARγ, Vitamin D receptor and RXRs), which are important for remyelination [39,40,41,70]. Moreover, transcription factors like Myrf, Olig1, Ascl1 and Olig2 that regulate developmental myelination also promote remyelination. For instance, Ascl1 expression increases during remyelination and is required for the proper timing of OPCs differentiation in demyelinated lesions [71]. Olig2 overexpression enhance OPC differentiation and accelerate remyelination [72]. Moreover, in mice lacking Olig1, oligodendrocytes fail to remyelinate and confirm the implication of this factor for efficient remyelination in the brain [73].

Recent single cell RNAseq data indicate that OPCs and oligodendrocytes are heterogeneous, suggesting presumably distinct functional states. For instance, single-cell RNAseq performed on oligodendroglial cells during mouse brain development as well as in the adult, revealed six sub-populations of myelinating oligodendrocytes that are transcriptionally distinct, suggesting that oligodendrocytes are functionally heterogeneous in different CNS regions [74]. Intriguingly, this heterogeneity is also found in the adult mouse CNS following demyelination and in MS lesions [75] (see next chapter). In the experimental encephalomyelitis mouse model of demyelinating, scRNAseq experiments showed that oligodendroglial cells can express immune specific genes such as MHCI and MHCII and that OPCs can have phagocytic and antigen presenting functions [75]. These data highlight unexpected immunomodulatory functions of oligodendroglial cells, which are commonly attributed to macrophages/microglia. Importantly, oligodendroglia heterogeneity is also seen in MS brain tissue, both in lesions and normal appearing white matter, with seven sub-clusters of OLs, some of which showed similarities with those described in the mouse CNS [76]. Single nuclear RNAseq of oligodendroglial cells in MS lesions also revealed that mature OLs upregulate myelin gene transcripts, arguing that mature OLs, rather OPCs, could contribute to remyelination in the human brain, as also pointed by recent findings using retrospective carbon 14 cell fate mapping [77]. These recent studies also challenge our current view indicating that OPCs are the main contributing cell type to remyelination and suggest instead that these cells could contribute to autoimmune-mediated demyelination in the human brain [78].

In several neurodegenerative diseases, CNS myelin can be damaged and in some cases the capacity of OL regeneration and remyelination are affected [79,80]. In the next chapter, we focused on MS, the most common chronic inflammatory demyelinating of the CNS in young adults. Different in vitro and in vivo experimental models have been used to understand the pathophysiology of this disease, in particular oligodendrocyte differentiation and remyelination failure that contribute to MS progression.

## 3. Toward Remyelination and Repair in Multiple Sclerosis (MS)

### 3.1. MS: Clinical Forms and Pathological Features of the Lesions

MS is the most frequent chronic inflammatory disease associated with CNS demyelination. There are more than 2 million people affected in the world. MS is most prevalent in Europe, North America and Australia while it is lower in Asia and Africa, and usually begins in early adulthood. This autoimmune disease is characterized by diffuse inflammatory demyelinated lesions in several CNS region including white and gray matter, brain stem, spinal cord and optic nerve [81]. Four main clinical forms have been described in MS. The most common one is the relapsing–remitting form (RRMS) affecting about 80% of MS patients, and which is characterized by symptoms over a period of several days followed by stabilization and then complete or partial recovery. Persistent signs after several years can increase and RRMS may progress between relapses to a secondary progressive MS form (SPMS). The other group of affected patients (20%) develops, from the onset of the disease, gradually progressive and severe clinical symptoms (primary progressive MS, PPMS). The overlap of PPMS and SPMS represents clinical symptoms of progressive relapsing MS (PRMS). Patients of this group present a progressive worsening of the condition from the beginning, similar to PPMS, accompanied with occasional relapse episodes of intensified clinical symptoms similar to those observed in relapsing-remitting MS (RRMS).

Several types of disseminated MS lesions have been characterized and classified according to the presence or absence of inflammatory cells, composed mainly of macrophages and microglia. In active demyelinating lesions, a dense infiltration of macrophages that phagocytes myelin products is observed. Chronic active and smoldering MS lesions (mixed active/inactive lesions) are characterized by the presence of activated macrophage/microglia within or surrounding demyelinated lesions, respectively, and myelin degradation products in phagocytes [82]. Only few macrophages/microglia cells are found, in the center of chronic active of demyelinated lesions. Chronic active lesions are characterized by a hypocellularity in the lesion core and may show thin myelin sheaths at the edge, representing remyelination. Inactive lesions are hypocellular in the lesion area with a drastic reduction of macrophages/microglia cells compared to the other types of MS lesion, and almost completely depleted of mature oligodendrocytes [83]. Shadow plaques are sharply demarcated plaques in a typical MS distribution, associated with only a modest decrease of axonal density. Shadow plaques represent complete remyelination of previously demyelinated plaques with disproportionately thin myelin sheaths and the presence of few macrophages. Demyelination may be associated with the loss of oligodendrocytes, and block of OPC differentiation to mature oligodendrocytes [84]. In this case, remyelination is insufficient, and chronic demyelination contributes to axonal loss, which lead to progressive and irreversible neurological disability.

Heterogeneity of MS lesions, as well as the unknown etiology and events that initiate this disease complicate the generalization of therapy in all MS patients. Two pathological hypothesis, which could trigger CNS demyelination, were proposed by the scientific community. The first one is called “outside-in”. In this case, immune cells of peripheral adaptive immune system migrate across the blood brain barrier enter and attack the CNS after their activation, expansion and secretion of pro-inflammatory cytokines. This inflammation state stimulates microglia and astrocytes and recruits other immune cells that amplify inflammatory response and induce the destruction of myelin, oligodendrocytes and axons. The “inside-out” mechanism suggests that this attack starts inside the CNS. Degeneration of oligodendrocytes and myelin is the initial event, and after oligodendrocyte death and myelin degradation, a secondary autoimmune attack is initiated, resulting in inflammatory demyelination [85].

### 3.2. Therapeutic Approaches in MS

#### 3.2.1. Immunomodulatory Therapy

Currently, all treatments approved for MS therapy aim to modulate the immune response with immunomodulatory and/or immunosuppressive drugs. These therapeutic strategies are frequently used to treat clinically significant relapses. All these disease-modifying therapies are used to modulate or suppress inflammation. Several drugs have been tested so far in clinical practice, some of which can reduce relapses and symptoms in MS patients. More than 20 drugs approved by the Food and Drug Administration (FDA) are used for RRMS, and some of them are used in the secondary progressive forms of MS, when still associated with relapsing-remitting phases. This list of FDA approved drugs includes interferon β-1a/1b, mitotranxone, glatiramer acetate, dimethyl fumarate, teriflunomide, fingolimod, siponimod, cladribine, ocrelizumab, ofatumumab, alemtuzumab, daclizumab, natalizumab and ozanimob [86].

#### 3.2.2. Remyelination Therapies

Most of the current immunomodulatory therapies used in MS fail to prevent or reverse progressive forms of the disease. This support the notion that persistent demyelination may, in some types of lesion, be the major cause of neurological disability in patients. The current challenge in MS, in addition to therapies targeting inflammation, is to promote remyelination in order to prevent axonal degeneration and, therefore, accumulation of neurological disability. Several promising ongoing research strategies for drug discovery of pharmacological compounds target OPC differentiation with the ultimate goal to find new treatments for progressive MS. Although, it is worth noting that human oligodendrocytes, derived from IPSCs of MS patients, have normal myelination properties and interact with axons when grafted in the *shiverer:rag2* mouse brain [87], suggesting that the oligodendrocyte differentiation block is not related to an intrinsic defect of this cell type.

**Figure 2:**
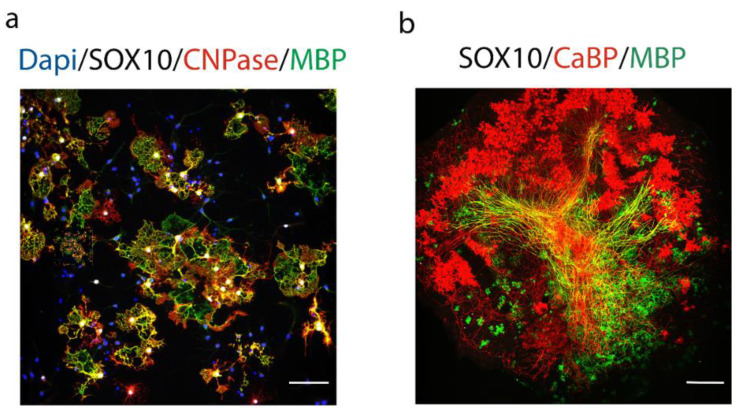
In vitro and ex-vivo models used in screening and validation assays of compounds affecting myelination. (**a**) Oligodendrocyte differentiation in primary rat OPCs culture. OPCs cells are obtained from P1 neonatal rat cortices and differentiated 5 days in vitro into mature oligodendrocytes expressing Sry-related HMg-Box gene 10 (Sox10, white), 2′,3′-cyclic nucleotide 3′-phosphodiesterase (CNPase, red) and myelin basic protein (MBP, green). Nuclei are counterstained with 4′,6-diamidino-2-phénylindole (Dapi) (bleu). (**b**) Organotypic cerebellar slices maintained in culture for 10 days to visualize ex-vivo myelination. Sox10 was used to stain oligodendroglia (white), MBP for myelin (green) and calbindin (CaBp) for Purkinje cells (red). Scale bars (a: 80 µm, b: 160 µm).

The development of remyelination therapies in patients with MS requires profound knowledge of the cellular and molecular mechanisms regulating myelination, in order to find potential pharmacological targets modulating this process, which could be tested under demyelinating conditions (hypothesis-driven approach). The other approach is to perform a large screen of chemical libraries with the goal to find drugs enhancing OPC differentiation and remyelination, using for example primary rat OPCs culture and/or organotypic cerebellar slice cultures (Figure 2). The development of new drugs stimulating oligodendrocyte’s maturation and myelination represent a promising approach, which can be used alone or in combination with immunomodulatory therapy.

Several compounds favoring remyelination and targeting several signaling pathways have been identified in the last decade. In 2013, Deshmukh et al. [88] performed a high content screening for MBP expression using primary rat OPCs and identified benztropine among the most effective compounds. Benztropine acts as an anti-muscarinic receptor M1/M3 and is a FDA approved treatment for Parkinson’s disease. This compound increases MBP expression in rat primary OPCs derived from optic nerves, induce remyelination and decrease significantly clinical symptoms in the experimental autoimmune encephalomyelitis (EAE) model of MS, when administered alone or in combination with approved immunosuppressive treatments for MS, interferon -β and FTY720. Benztropine enhances OPC differentiation and remyelination in vivo in the cuprizone-induced model of demyelination. Clemastine, another anti-muscarinic was identified among a cluster of anti-muscarinic compounds including benztropine, quetiapine and others, using a micropillar array high throughput screening for OPC differentiation and myelination. Clemastine and benzatropine are FDA-approved compounds that cross the blood-brain barrier. They were also the most effective drugs in enhancing oligodendrocyte differentiation and myelination, using purified oligodendroglia cultured alone or with purified DRG neurons. Clemastine enhances and accelerates the kinetics of remyelination in mice after lysolecithin-induced demyelination [89]. M1 muscarinic acetylcholine receptor was identified as a target for remyelination and its oligodendroglial-specific genetic ablation accelerates remyelination and prevents axonal loss in mouse EAE [90]. In addition, clemastine rescues behavioral changes by enhancing remyelination in the cortex and corpus callosum in the cuprizone model of demyelination [91], and in the prefrontal cortex of adult mice following social isolation [92]. Altogether, these findings demonstrate that enhancing remyelination supports axonal integrity and neuronal function in several experimental models of MS.

In addition to muscarinic receptors, LINGO-1 is expressed by oligodendrocytes and acts as a potent negative regulator of OLs differentiation and myelination. Loss of LINGO-1 function using LINGO-1 RNA-mediated interference (RNAi), dominant negative LINGO-1, or soluble human LINGO-1 (LINGO-1-Fc) decreases RhoA activity, which has been implicated in OLs differentiation and axonal myelination [93]. Opicinumab (BIIB033), has been recently developed, as a CNS-specific membrane glycoprotein that acts as a LINGO-1 blocker. The targeted inhibition of LINGO-1 acts by blocking molecules such as Nogo-A and RhoA, and consequently promote remyelination and axonal protection [94,95]. In a randomized, double-blind and placebo-controlled, dose-ranging phase II study (SYNERGY) (NCT01864148), opicinumab was used in combination with intramuscular Interferon-beta (IFNβ)-1a therapy. The results did not show a significant dose-linear improvement in disability, but some subpopulation identified in the study seems to respond most to opicinumab therapy [96]. To further support these data, the phase 2 AFFINITY study (NCT03222973) of opicinumab, as an add-on to multiple anti-inflammatory disease-modifying treatments, has been initiated to assess the efficacy and safety of this drug in a targeted population of patients with relapsing MS identified from the SYNERGY study.

GSK239512 and GSK247246 are histamine H3 receptor (H3R) antagonists that have been developed for the treatment of Alzheimer’s disease and schizophrenia [97]. GSK247246 was identified as a potential remyelinating compound after the screening of 1000 compounds on rat OPC cultures. This compound enhances remyelination in the corpus callosum and cortex in the cuprizone model of demyelination model [98]. In a phase II, clinical trial as an add-on to a preexisting disease-modifying therapy with intramuscular interferon-β1a or glatiramer acetate including 131 patients (NCT01772199), treatment with GSK239512 shows a small positive effect on remyelination compared to placebo treatment, although some adverse events like insomnia were more common with in participants treated with GSK239512 [99].

Najm et al. [100], using a high-content imaging phenotypic screening, identified miconazole and clobetasol that act on OPCs through mitogen-activated protein kinase (MAPK) and glucocorticoid receptor (GR) signaling, respectively. These two drugs promote myelination in several in vitro and in vivo models and enhance the generation of oligodendrocytes from human OPCs in vitro. Later, the same group showed that a group of pro-myelinating small molecules, identified in the first study, inhibits CYP51 (cytochrome P450, family 51), TM7SF2 ((Transmembrane 7 Superfamily Member 2), or EBP (emopamil binding protein) and induces accumulation of 8,9-unsaturated sterols that drive oligodendrocyte differentiation and remyelination [101]. Clobetasol and halcinonide have also been shown to act as a smoothened agonist and activate retinoid X receptor γ (RXRγ) [102]. The use of mixed embryonic cortical cell cultures containing both OPCs and axons was used also to screen a FDA-approved library of 727 compounds and select 27 hit compounds promoting myelination [103,104]. These compounds were grouped according to their mechanism of action, such as muscarinic receptor antagonists, selective estrogen receptor modulators or adrenergic agonists.

The number of myelinating compounds identified and tested in pre-clinical or clinical trials has significantly increased over the past 5 years with a deeper understanding of the mechanisms regulating the development of oligodendrocytes and the myelination process. The variety of screening strategies using various in vitro culture systems and rodent models of MS allowed the most efficient drugs to be selected for further clinical trials (Table 1). However so far, none of the listed drugs have reached clinical use in MS. The discovery and development of new promyelinating drugs is still a major unmet medical need to prevent disease progression in MS.

## 4. Conclusions

Deciphering mechanisms implicated in OL differentiation, myelination and remyelination is a major advance to understand their implication in neurodegenerative diseases. The use of different in vitro and in vivo physiopathological models aims to better understand regulation and deregulation of this major glial cell type, with the aim to identify the best therapeutic targets for OL regeneration and remyelination. In MS, OLs are the main actors and are essential to effectively remyelinate lesions in patients. In addition to immune-based strategy, the investigation of potential promyelinating agents is currently the aim of several promising ongoing studies. The combination of these two strategies to modulate the immune response and improve myelin repair in patients with MS could be complementary for the development of effective regenerative therapies.

## Figures and Tables

**Table 1:** Clinical trials results related to remyelination.

Drugs	Mechanism of Action	Clinical Trial	Efficacity in Clinical Trial	ClinicalTrials.gov Identifier
Biotin or MD1003	Cofactor for carboxylases involved in fatty acid synthesis	Phase III: patients suffering from progressive MS	MD1003 cannot be recommended for PMS	NCT02936037 NCT03215433 NCT03302806 NCT03552211
Adrenocorticotropic hormone (ACTH)	Polypeptide tropic hormone	Phase IV: patients with RRMS or SPMS with new contrast-enhancing lesions	Ongoing	NCT02446886
Bazedoxifene Acetate	Selective estrogen receptor modulator	Phase II: patients with RRMS	Ongoing	NCT04002934
BIIB061	Anti-tau mAb	Phase II: patients with RMS	Ongoing	NCT04079088
Clemastine	Antimuscarinic/antihistamine	3 Phase II: patients with acute optic neuritis/patients with relapsing forms of MS	Reduced latency delay of VEPs	NCT03109288 NCT02521311 NCT02040298
Nanocrystalline gold	Increase levels of the NAD+, intracellular ATP levels and extracellular lactate levels	Phase II: patients with chronic vision impairment as a result of RRMS	Ongoing	NCT03536559
Domperidone	Dopamine antagonist	2 Phase II: RRMS patients who are being treated with standard DMT and have new lesions/SPMS	No results posted	NCT02493049 NCT02308137
RHIgM22	Remyelinating monoclonal antibody	2 Phase I: RMS subjects/patients with all clinical presentations of MS	No results posted	NCT02398461 NCT01803867
Thyroid hormone	Heterodimers with retinoid X receptors	Phase I: subjects with MS	Short-term safety and tolerability in people with MS	NCT02760056 NCT02506751
Opicinumab (BIIB033)	Anti-LINGO-1 mAb	4 phase II trials: unilateral acute optic neuritis/active RMS used with Avonex	No effect on remyelination (ITT population)/ongoing	NCT01721161 NCT02657915 NCT01864148 NCT03222973
GSK239512	Histamine H_3_ receptor antagonist/antimuscarinic	Phase II: subjects with RRMS, receiving intramuscular interferon-β1a or glatiramer acetate	Positive effect sizes in MTR for GdE and Delta-MTR lesions	NCT01772199
Quetiapine	Dopamine type 2 and serotonin 2A (5HT2A) receptors antagonist/antimuscarinic	Phase I/II: subjects with RRMS	No results posted	NCT02087631
VX15/2503	Inhibition of semaphorin 4D	Phase I	Safe and well tolerated	NCT01764737
Testosterone	Binding to and activation of the androgen receptor	Phase II: patients with RRMS	Ongoing	NCT03910738
Olesoxime	Cholesterol-like neuroprotective compound	Phase I: patients with stable RRMS, on top of Interferon beta	No results posted	NCT01808885

VEP: Visual Evoked Potential; MTR: Magnetization Transfer Ratio; DMT: Disease-Modifying Treatment; GdE: Gadolinium-enhanced; mAb: Monoclonal Antibody; NAD: Nicotinamide Adenine Dinucleotide; ATP: Adenosine Triphosphate; MS: Multiple Sclerosis; RRMS: Relapsing–Remitting Multiple Sclerosis; SPMS: Secondary Progressive Multiple Sclerosis; PMS: Progressive Multiple Sclerosis; RMS: Relapsing Multiple Sclerosis; ITT: intention-to-treat.

## Data Availability

Not applicable.
